# Differential DNA methylation and CTCF binding between the ESR1 promoter a of MCF-7 and MDA-MB-231 breast cancer cells

**DOI:** 10.1007/s11033-023-09171-0

**Published:** 2024-01-18

**Authors:** Edén Víctor Montes-de-Oca-Fuentes, Karina Jácome-López, Anaís Zarco-Mendoza, Georgina Guerrero, José Luis Ventura-Gallegos, Sergio Juárez-Méndez, Alberto Jose Cabrera-Quintero, Félix Recillas-Targa, Alejandro Zentella-Dehesa

**Affiliations:** 1 Departamento de Medicina Genómica y Toxicología Ambiental, Instituto de Investigaciones Biomédicas, Universidad Nacional Autónoma de México, Ciudad de México, 04510 México; 2 Programa de Investigación en Cáncer de Mama, Instituto de Investigaciones Biomédicas, Universidad Nacional Autónoma de México, Ciudad de México, 04510 Mexico; 3 Unidad de Bioquímica, Instituto Nacional de Ciencias Médicas y Nutrición Salvador Zubirán, Ciudad de México, 14080 México; 4 División de Investigación Básica, Laboratorio de Virus y Cancer, Secretaría de Salud, Instituto Nacional de Cancerología, Ciudad de México, 14080 México; 5 Departamento de Biología Molecular y Biotecnología, Instituto de Investigaciones Biomédicas, Universidad Nacional Autónoma de México, Ciudad de México, 04510 México; 6 Departamento de Genética Molecular, Instituto de Fisiología Celular, Universidad Nacional Autónoma de México, Ciudad de México, 04510 México; 7 Laboratorio de Oncología Experimental, Secretaría de Salud, Instituto Nacional de Pediatría, Ciudad de México, 04530 México; 8 Red de Apoyo a la Investigación, Universidad Nacional Autónoma de México‑Instituto Nacional de Ciencias Médicas y Nutrición Salvador Zubirán, Ciudad de México, 14080 México; 9 Cancer Center, American British Cowdray Medical Center, Ciudad de México, 01120 Mexico

**Keywords:** CTCF, ESR1, TNBC, DNA methylation, Breast cancer

## Abstract

**Background:**

ESR1 is expressed by 60–70% of breast tumours. it’s a good prognosis factor and the target of hormone therapy. Optimization of ESR1 reactivation therapy is currently ongoing. Here we probe if the transcription factor CTCF plays a role in the differential expression of ESR1 in the breast cancer cell lines MCF-7 (ESR1+) and MDA-MB-231 (ESR1-).

**Methods and results:**

Knockdown of CTCF in MCF-7 resulted in decreased ESR1 gene expression. CTCF binds to the promoter of ESR1 in MCF-7 but not in MDA-MB-231 cells. CTCF ESR1 binding sites are unmethylated in MCF7 but methylated in MDA-MB-231 cells.

**Conclusion:**

ESR1 expression in MCF7 cells is dependent on CTCF expression. CTCF can bind to specific regions of the promotor of ESR1 gene in MCF-7 cells but not in MDA-MB-231 cells, this correlates with the methylation status of these regions and could be involved in the transcriptional regulation of ESR1.

**Supplementary Information:**

The online version contains supplementary material available at 10.1007/s11033-023-09171-0.

## Introduction

Why 30–40% of breast tumours lack ESR1 expression is not fully known. Mutations responsible for its subexpression have not been found [1]. Some studies have shown a good correlation between decreased ESR1 expression and DNA methylation of its promoters [2–4]. Here, via experiments in vitro with MCF-7 cells (ESR1+) we propose that the methylation status of the high CpG island near promoter A of ESR1 is involved in the binding affinity of *CTCF to this locus and may be involved in the regulation of ESR1’s expression* regulation.

## Materials and methods

### Cell culture

The MCF-7 and MDA-MB-231 cell lines were obtained from the ATCC and cultured in *RPMI 1640* (Gibco, USA) supplemented with 10% fetal bovine serum (FBS) (Gibco, USA) and 1% penicillin/streptomycin (Gibco, USA) at 37 °C in a 5% CO_2_ incubator. These cell lines don’t appear in the list of commonly misidentified cell lines (International Cell Line Authentication Committee). The experiments were done with cells in passage number 10 or less. We routinely compared the morphology of our cells to those reported by the ATCC and didn’t see outstanding discrepancies. The authenticity of the MCF-7 and MDA-MB-231 cell lines used in this study can be verified by the certificates of analysis that appear in Online Resource 1 and 2, respectively.

### MCF-7 transduction with shCTCF

MCF-7 cells were transduced as described previously [5] with lentiviral particles containing a shRNA targeting CTCF kindly donated by FRT.

### RT-PCR analysis

Total *RNA* from the cell lines was extracted with TRIzol (Invitrogen, USA). *cDNA* was synthesized using *RNA*, reverse transcriptase, and oligo dT_18_ (Promega (Madison, USA). *PCR* amplification was done using GoTaq Flexi DNA Polymerase (Promega, USA). The primers used for PCR amplification are listed in Online_resource_3. All primers were exon-exon primers.

### Western blot analysis

Total protein was extracted using *RIPA* buffer, and 40 µg of protein was separated on a 7% polyacrylamide gel (by SDS-PAGE). The proteins were transferred from the gel to polyvinylidene fluoride (*PVDF*) membranes (Bio-Rad, USA) using a Trans-Blot Cell System (Bio-Rad, USA). The membranes were probed with a 1:2000 dilution of anti-*CTCFα* (Millipore, USA) and a 1:15,000 dilution of anti-*β-tubulin* (Santa Cruz Biotechnology, USA) antibodies. The membranes were incubated with a 1:10,000 dilution of HRP-conjugated anti-immunoglobulin. The signals were detected by enhanced chemiluminescence using Pierce Fast SuperSignal *ECL* Western Blotting Substrate (Thermo Fisher Scientific, USA). Densitometry analysis was performed using ImageJ software (https://imagej.nih.gov/ij/).

### Microarray processing

Microarray processing was performed according to the technical specifications of GeneChip 1.0 (Affymetrix, USA). Data analysis was achieved using CEL files by means of Genomics Suite v6.6 software (Partek, USA). Bioinformatics analysis was performed according to previously described methods [6]. Principal Component Analysis (PCA) was used to group and categorize the data. Differentially expressed genes were detected with ANOVA using mock-transduced cells as reference controls. Moreover, the shRNA-transduced and mock-transduced cells were compared by means of a geometric least-squares means model, and the significant genes were clustered based on the Euclidean method by means of average linkage.

### DNA isolation and DNA methylation analysis

*Total DNA* extracted from the cell lines was modified using a sodium bisulphite PCR protocol [5]. The DNA fragments of interest were *PCR*-amplified using the primers listed in Online_resource_3, cloned into the *pGEM-T Easy System* (Promega), and sequenced using SP6 or T7 primers. DNA methylation primers were designed using Methyl Primer Express, MethPrimer software and OligoCalc based on the sequence of ESR1. The positions were assigned based on the + 1 site in the 5’ UTR of isoform A, as described previously [7].

### Chromatin immunoprecipitation assay (ChIP)

Assays were performed as described previously [5]. The chromatin solutions were incubated with or without 4 µg of anti-CTCF or anti-acH3K4 antibodies (Millipore, USA). DNA was analysed by PCR using specific primers. CTCF1Fw and CTCF1Rev were used for binding site 1 (CTS1), CTCF2Fw and CTCF2Rev were used for binding site 2 (CTS2), in the ESR1 gene (Online_resource_3). As negative control, primers specific to exon 27 of the Rb gene (Ex27RBF2 and Ex27RBRev) were used [8].

## Results and discussion

### CTCF-associated gene expression profile

**Fig. 1:**
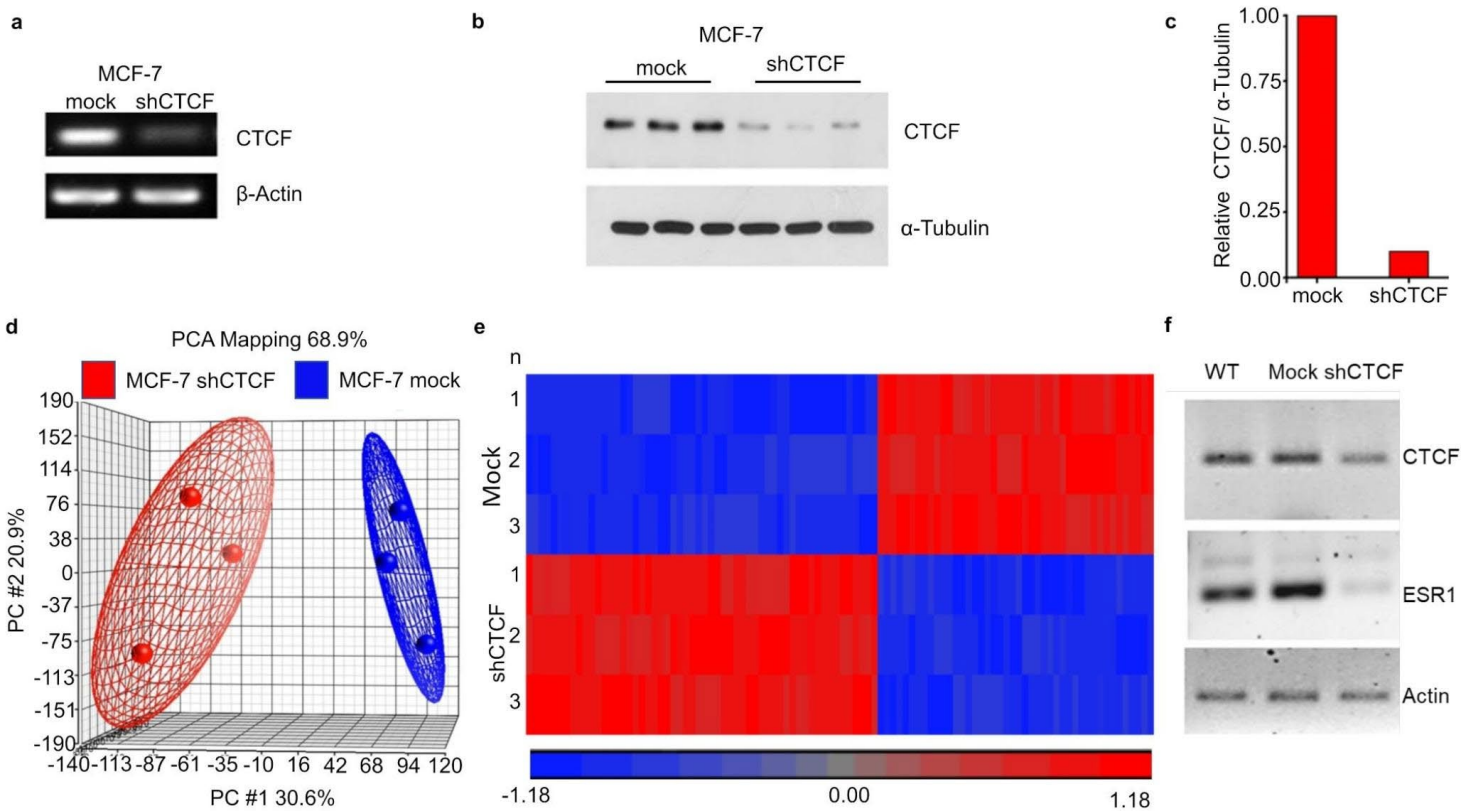
Expression profile of shCTCF MCF7 cells and mock transduced cells. **a** RT-PCR of CTCF in MCF-7 cells transduced with shCTCF or not (mock). Β-actin was used as a loading control. **b** Western blot of CTCF in MCF-7 cells transduced with shCTCF or not (mock). α-Tubulin was used as a loading control. **c** Densitometry analysis of the western blot in **b**. **d** PCA analysis to compare the intragroup and intergroup variations in the general gene expression profiles of MCF-7 cells transduced with shCTCF or not (mock). Each circle represents a biological replica. **e** Heat map representation of the hierarchical clustering analysis of the 78 differentially expressed genes (columns) between MCF-7 cells transduced without (mock) or with shCTCF. Each row represents an independent experiment. The 36 downregulated genes and the 42 upregulated genes between the experimental conditions are depicted in hues of blue and red, respectively. **f** RT-PCR of CTCF and ESR1 in wild type (WT), mock transduced or shCTCF transduced MCF7 cells. Actin was used as a loading control

We sought to determine the target genes of CTCF in MCF-7 cells. For that purpose, such cells were transduced with a shCTCF. This reduced CTCF’s mRNA (Fig. 1a) and protein levels (Fig. 1b-c) in comparison to mock transduced cells (7 days post-transduction).

Afterwards, we extracted total RNA from shCTCF- and mock-transduced MCF-7 cells and analysed it on a Gene Chip 1.0 microarray. General gene expression was visualized according to the transduced groups by means of principal component analysis (PCA). The analysis showed similar intragroup gene expression profiles, indicating that these data sets have the power to discriminate both mock- and shCTCF-transduced cells (Fig. 1d). After that, we performed a comparative analysis, and we used mock transduced cells as a baseline to determine significant differences in gene expression in the transduced cells. The significantly differentially expressed genes are clustered in the heat map in Fig. 1e. The ESR1 gene was one of the downregulated genes identified in the microarray analysis with a *p value* = 0.001. By RT-PCR we corroborated that compared to mock cells, shCTCF cells had lower levels of CTCF and ESR1 (Fig. 1f). Our findings suggest that ESR1 expression can be directly regulated by CTCF. A limitation of our study is that ESR1 proteins levels were not evaluated in the shCTCF-transduced MCF-7 cells. Also, in future studies CTCF should be silenced and overexpressed in other ESR1 + and ESR1- cell lines, respectively.

### CTSs in the ESR1 gene

**Fig. 2:**
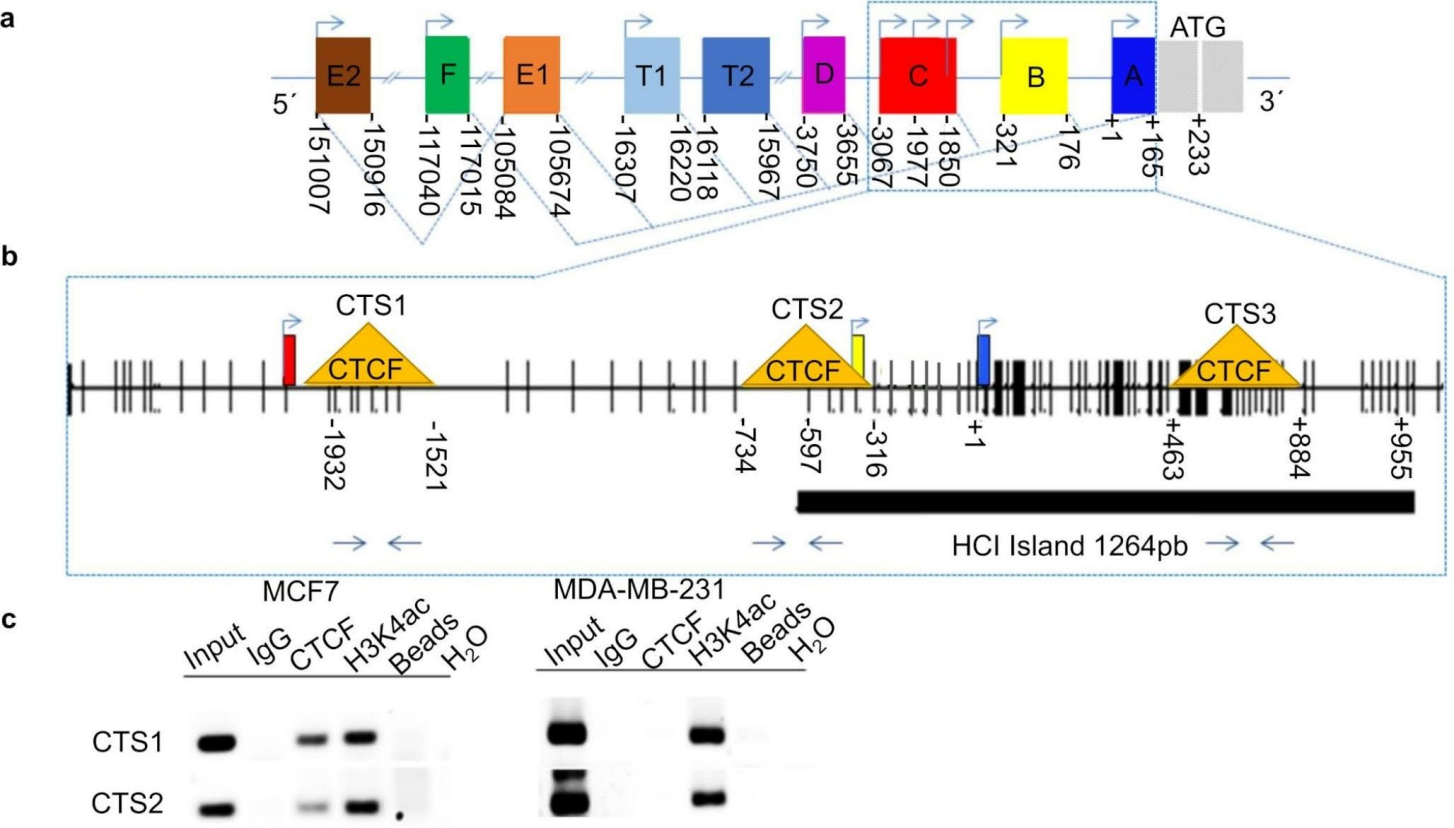
CTCF binds to two sites in the ESR1 gene. **a** ESR1 genomic domains showing the TSSs of the promoters and first exon. **b** ESR1 genomic domains showing the putative CTSs as yellow triangles and the CpG sites as black vertical lines. The many CpG sites (~ -597 to + 955) in ESR1 show the presence of an HCI, which is susceptible to DNA methylation. **c** CTCF ChIP in MCF-7 and MDA-MB-231 cells at CTS1 and CTS2. H3K4ac ChIP was performed in the same loci to evaluate for heterochromatin and euchromatin

Henceforth, we investigated whether CTCF directly binds the promoter of ESR1. We first evaluated *in silico* the sequence of the ESR1 gene (Fig. 2a) to identify DNA binding sites for CTCF (CTSs). We used human data in the UCSC Genome Browser [9], the CTCF ChIP-Seq databases [10] and Kim et al. [11]. Our analysis showed three possible CTSs (CTS1, CTS2 and CTS3) enriched within 1.8 kb of the ESR1 gene (Fig. 2b).

To confirm that CTCF bound in vivo to the CTSs of ESR1, we used *ChIP* assays. As depicted in Fig. 2c, in MCF-7 cells CTS1(-1899 to -1687) was the best site for binding of CTCF to the ESR1 promoter, although CTS2 (-683 to -437) also showed a signal. In contrast in MDA-MB-231 cells CTCF wasn’t bound to any of the CTSs. Our study in this aspect is also limited by the fact that we only analysed ChIP data of one ESR1 + and one ESR1- cell line. In the future ChIP data from more ESR1+/- breast cancer patients should be analysed to ponder the role of CTCF and of other transcription factors, protein cofactors or even of non-coding RNAs for example.

### CTFC and DNA methylation in the ESR1 gene

**Fig. 3:**
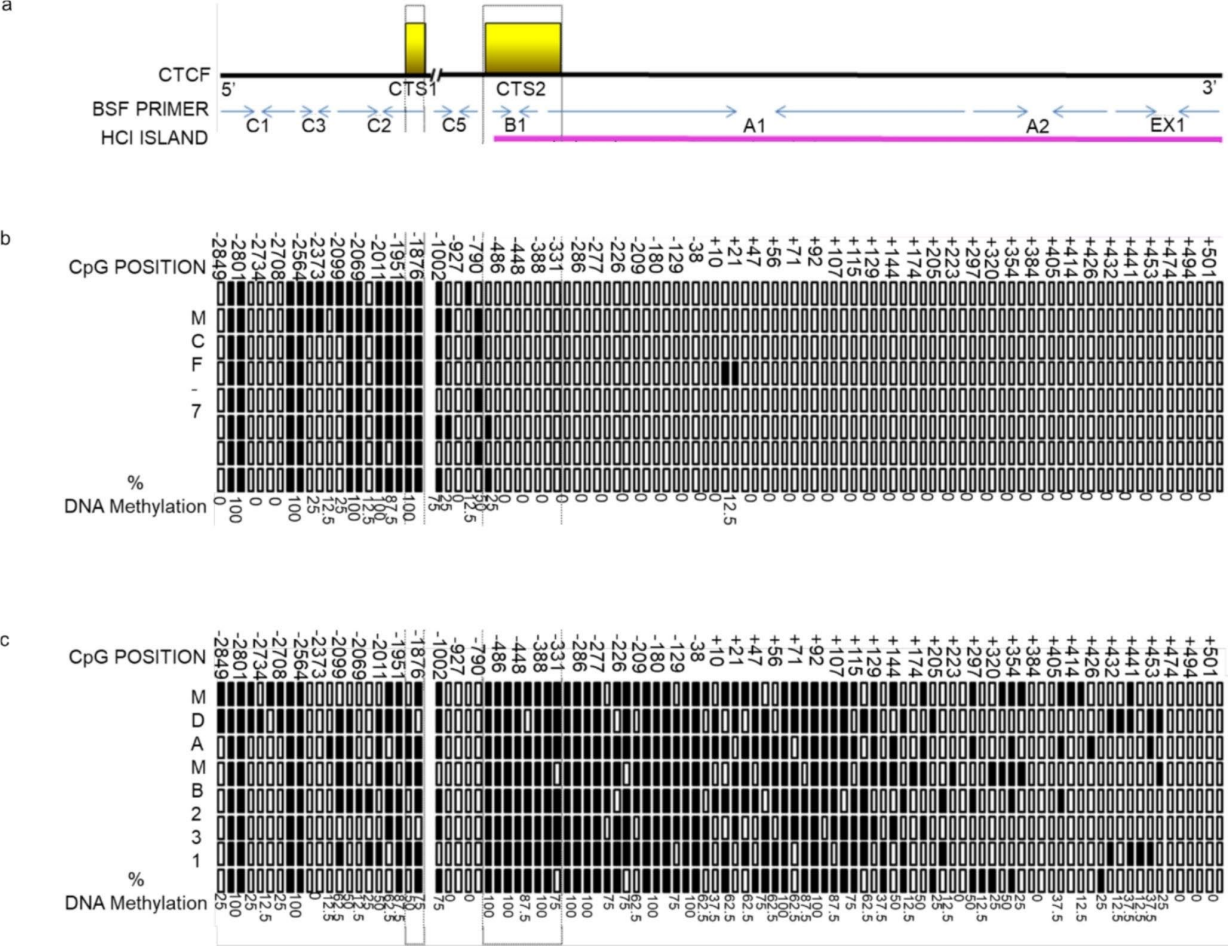
DNA methylation status of the ESR1 gene promoter in MCF-7 and MDA-MB-231 cells. **a** Schematic diagram showing as yellow rectangles, CTCF’s binding sites (CTS1 & 2); as blue arrows, the binding sites of the primers that were used to amplify these loci and as a pink rectangle, the location of the HCI. **b** MCF-7’s DNA methylation status, in the section of the ESR1 gene promoter where CTS1 & 2 are located. Each row represents a technical replica, and each column is a specific CpG. Methylated CpGs are filled in black and unmethylated ones are not. The % of methylation in each CpG is indicated at the bottom of each column. **c** Same as b but for MDA-MB-231 cells

To investigate whether *CTCF* could be a boundary that regulates *ESR1* gene transcription, we tested *DNA* methylation on high-CpG islands (HCIs) and CTSs in this gene (Fig. 3).

Our results showed differential methylation associated with the presence of *CTCF* in specific regions in the ESR1 promoter. As expected, *MDA-MB-231* cells had diverse patterns of methylation in the ESR1 promoter, while MCF-7 cells had a consistently unmethylated *ESR1* gene flanked by regions of dense *DNA*, suggesting that *CTCF* could be a methylation boundary that regulates the transcriptional activity of *ESR1.*

Our findings are in accordance with the fact that treatment with methyl transferases inhibitors, histone deacetylases inhibitors, glucocorticoids or vitamin D analogs have been able to re-activate the expression of ESR1 in MDA-MB-231 and other ESR1- cell lines. Such reactivation has rendered the cells sensitive to drugs that inhbit ESR1 and induced cell death [12]. Nevertheless, such drugs are not specifically reactivating ESR1 and could reactivate other genes that may increase the aggressiveness of the breast cancer cells. Future studies should identify the other reactivated genes and perhaps determine factors that can exclusively reactivate ESR1.

## Conclusion

ESR1 expression in MCF7 cells is dependent on CTCF expression. CTCF can bind to specific regions of the promotor of ESR1 gene in MCF-7 cells but not in MDA-MB-231 cells, this correlates with the methylation status of these regions and could be involved in the transcription regulation of the ESR1.

## Electronic supplementary material

Below is the link to the electronic supplementary material.

Online Resource 1

Online Resource 2

Online Resource 3

## Data Availability

Data supporting the findings of this study are available within the paper, or can be requested to the authors. All primer sequences used in this study are on the file Online_resource_1.pdf on the online version of this paper.
