# Supplementary material for: Differential DNA methylation and CTCF binding between the ESR1 promoter a of MCF-7 and MDA-MB-231 breast cancer cells
Source: Mol Biol Rep. 2024 Jan 18;51(1):148. doi: 10.1007/s11033-023-09171-0 (PMC10796618; doi:10.1007/s11033-023-09171-0)
Supplement: Supplementary file 3 — Online Resource 3 [file 11033_2023_9171_MOESM3_ESM.pdf]

Molecular Biology Reports

Differential DNA methylation and CTCF binding between the ESR1 promoter A of MCF-7 and MDA-MB-231 breast cancer cells

Edén Víctor Montes-de-Oca-Fuentes, Karina Jácome-López, Anaís Zarco-Mendoza, Georgina Guerrero, José Luis Ventura-Gallegos, Sergio Juárez-Méndez, Alberto Jose Cabrera-Quintero, Félix Recillas-Targa and Alejandro Zentella-Dehesa[1-5 *]

1. Departamento de Medicina Genómica y Toxicología Ambiental, Instituto de Investigaciones Biomédicas, Universidad Nacional Autónoma de México, 04510 Ciudad de México, México
2. Programa de Investigación en Cáncer de Mama, Instituto de Investigaciones Biomédicas, Universidad Nacional Autónoma de México, 04510 Ciudad de México, Mexico
3. Unidad de Bioquímica, Instituto Nacional de Ciencias Médicas y Nutrición Salvador Zubirán, 14080 Ciudad de México, México
4. Red de Apoyo a la Investigación, Universidad Nacional Autónoma de México-Instituto Nacional de Ciencias Médicas y Nutrición Salvador Zubirán, 14000 Ciudad de México, México
5. Cancer Center, American British Cowdray Medical Center, 01120 Ciudad de México, Mexico

*Corresponding author: Alejandro Zentella-Dehesa, azentell@biomedicas.unam.mx

Online Resource 3. Primers used in this report.

Name	Sequence	Region	Frag. Pb
DNA methylation			
ESR-C1 Fw	5´-GGT GTT ATG TGT TTT GGG ATT TTG-3´	-2875	341
ESR -C1Rev	5´-CCA ACT AAA ATA TAA TAC TCC TCC ACT T-3´	-2534	
ESR -C2 Fw	5´-TTG TTT TTT GTG AGT AGA TAG TAA GTT TTT-3´	-2156	313
ESR -C2Rev	5´-TCA AAA CAA ACC TAC CCT ACT AAA TC-3´	-1843	
ESR -C3 Fw	5´-TTA ATT GAG TTA TTT ATG GAA AGG TTT GTG-3´	-2605	359
ESR -C3Rev	5´-CTA CTA CCA CCC CAA ACA AAA AAA A-3´	-2246	
ESR -C5 Fw	5´-TAT TTG GAT ATT TGA TTT TGT ATA TTT TAG-3´	-1043	280
ESR -C5Rev	5´-AAT TCA AAA AAA CCT TTA ATT AAA AC-3´	-763	
ESR -B1 Fw	5´-GGA AGA AAT TGA GGT TTT GGT AGG-3´	-635	272
ESR -B1Rev	5´-CTT TCT ATT TAA TTC CCC CTC-3´	-363	
ESR -A1 Fw	5´-CCT GAA GCT TGT TGT TAA ATA TAG TTG TTT GTG-3´	-585	571
ESR -A1 Rev	5-´CCT GAA GCT TAC CTC CAA CTT TAA ATA CTA ATC-3´	-14	
* ESR -A2 Fw	5´-GAT TAG TAT TTA AAG TTG GAG GTT-3´	-34	287
* ESR -A2Rev	5´-AAT ATA AAA AAT CAT AAT CAT AAT CC-3´	+254	
ESR -EXON1 Fw	5´-GTA TTT GGG ATG GTT TTA TTG TAT TAG-3´	+258	281
ESR -EXON1Rev	5´-CTA TTA AAT AAA AAA AAA CCC CC-3´	+538	
RT-PCR			
ERa RT-Fw	5´-AGA CAT GAG AGC TGC CAA CC-3´		299
ERa RT-Rev	5´-GCC AGG CAC ATT CTA GAA GG-3´		
CTCF RT-Fw	5´-GCC AGC AGG GAC ACA TAC AAG-3´		217
CTCF RT-Rev	5-´GCT TTC GCA AGT GGA CAC C-3´		
ActFw	5'-GTG GGG CGC CCC AGG CAC CA-3'		540
ActRev	5'-CTC CTT AAT GTC ACG CAC GAT TT-3'		
CHROMATIN INMUNOPRECIPITATION ASSAY (ChIP)			
CTCF1Fw	5'-CCC ATG GAA CAT TTC TGG AA-3'	-1899	212
CTCF1Rev	5'-GCA AAT GGC ACA AAG AAT CC-3'	-1687	
CTCF2Fw	5'-CTC AGA AGG TTG TGG AAG GGT-3'	-683	246

CTCF2Rev	5'-CAC TGT AAC CGA GAT AGG GCA-3'	-437	
Ex27RB Fw	5'-GGT ATG TAA CAG CGA CCG TGT G-3'		[1]
Ex27RB Rev	5'-CTC TTC CTT GTT TGA GGT ATC C-3'		

References

1. Soto-Reyes E, Recillas-Targa F (2010) Epigenetic regulation of the human p53 gene promoter by the CTCF transcription factor in transformed cell lines. Oncogene 29:2217–27. https://doi.org/10.1038/onc.2009.509
